# Application of UAV remote sensing for vegetation identification: a review and meta-analysis

**DOI:** 10.3389/fpls.2025.1452053

**Published:** 2025-05-30

**Authors:** Baohua Chang, Fei Li, Yuncai Hu, Hang Yin, Zhenhua Feng, Liang Zhao

**Affiliations:** ^1^ College of Resources and Environment, Inner Mongolia Agricultural University, Hohhot, China; ^2^ Inner Mongolia Key Laboratory of Soil Quality and Nutrient Resources, Key Laboratory of Agricultural Ecological Security and Green Development at Universities of Inner Mongolia Autonomous, Hohhot, China; ^3^ Precision Agriculture Laboratory, School of Life Sciences, Technical University of Munich, Freising, Germany

**Keywords:** unmanned aerial vehicle (UAV), remote sensing, meta-analysis, identification, classification, overall accuracy (OA)

## Abstract

Green vegetation is an essential part of natural resources and is vital to the ecosystem. Simultaneously, with improving people’s living standards, food security and the supply of forage resources have become increasingly the focus of attention. Therefore, timely and accurate monitoring and accurate and timely vegetation classification are significant for the rational utilization of agricultural resources. In recent years, the unmanned aerial vehicle (UAV) platform has attracted considerable attention and achieved great success in the application of remote sensing identification of vegetation due to the combination of the advantages of satellite and airborne systems. However, the results of many studies haven’t yet been synthesized to provide practical guidance for improving recognition performance. This study aimed to introduce the primary classifiers used for UAV remote-sensing vegetation identification and conducted a meta-analysis of relevant research on UAV remote-sensing vegetation identification. This meta-analysis reviewed 79 papers, analyzed the general characteristics of spatial and temporal distribution and journal sources, and compared the relationship between research objectives, sensor types, spatial resolution, research methods, number of target categories, and the overall accuracy of the results. Finally, a detailed review was conducted on how unmanned aerial vehicle remote sensing is applied in vegetation identification, along with the current unresolved issues and prospects.

## 1 Introduction

Vegetation, a crucial component of natural resources, serves as the collective term for surface plant communities (Sun et al., 2015). It is an essential component of natural resources and plays a vital role in the ecosystem (Pavol and Patrícia, 2017; Tran and Brown, 2018). It is not only the production base of agriculture and animal husbandry and environmental fundamental element for human survival (Jones et al., 2019), but also the basis of ecological security barrier protection (Xu et al., 2021), with ecological (Jin et al., 2020), production (Kessler et al., 2014) and life functions. However, in recent years, ecological problems have become more and more prominent due to natural factors such as rising temperature and human factors that pay attention to the production function of vegetation but ignore the ecological function (Jun Li et al., 2007; Kooch et al., 2022; Oñatibia et al., 2020), leading to ecological problems such as fragile vegetation ecosystem (Jiang et al., 2022), soil erosion (Fang et al., 2019), and reduced vegetation utilization performance (Liu et al., 2021b). Simultaneously, the problem of food security (Campi et al., 2021; Potgieter et al., 2013) and forage resource supply (Berauer et al., 2021) has become the hot spot of people’s attention. Therefore, the monitoring and high-quality identification data of vegetation growth processes directly impact the natural resource management and agricultural and animal husbandry industries, which are crucial for farm planning and management decisions.

Nevertheless, traditional ground investigation and identification methods have many limitations, such as extended time, low efficiency, labor-intensive, and destructive. In particular, some large-scale and frequent research is even more challenging to achieve, which brings much trouble to research and application (Wardlow and Egbert, 2008). It is even more to identify and manage the grassland vegetation, which is not easily controlled, and the individual plants are small. In contrast, remote sensing technology has irreplaceable advantages (Valasek et al., 2016). Remote sensing technology has recently gained significant attention from researchers and practitioners as a non-destructive and efficient method for monitoring vegetation. At present, agricultural remote sensing has been widely used in vegetation mapping (Prošek and Šímová, 2019), vegetation identification and classification (Heuschmidt et al., 2020), vegetation growth monitoring (Li et al., 2019a), crop yield estimation (Wang et al., 2021), vegetation pest monitoring (Cao et al., 2013), biomass estimation (Yan et al., 2015) or other fields and achieved good performance. Therefore, remote sensing tools also play a crucial role in relevant identification tasks in the vegetation field by collecting spectral data (Li et al., 2021; Sun et al., 2018; Tao et al., 2018).

Remote sensing data are mainly collected through three platforms, namely spaceborne, airborne and terrestrial platforms. Satellite remote sensing data has the advantages of covering a wide range and comprehensiveness being comprehensive. Nevertheless, the temporal resolution of satellite remote sensing data with high spatial resolution is generally low, so vegetation phenology cannot be directly extracted. The spatial resolution of medium/low-resolution data alone is insufficient to capture the distinctive characteristics of vegetation, making it challenging. In contrast, the airborne platform not only has higher flexibility but also higher economic value. It can obtain flexible remote sensing image resolution (Yang et al., 2014). In particular, unmanned aerial vehicle (UAV) platforms have aroused great concern in agricultural remote sensing identification applications because they combine the respective advantages of satellite and airborne systems (Hunt et al., 2010; Padró et al., 2019), such as fast response capability, high image resolution, and low operating costs (Colomina and Molina, 2014; Pádua et al., 2017).

With the increasing universality of UAV remote sensing data, research on exploiting the conveniences of the information, it provides to study agriculture monitoring and management is gaining considerable pace. More and more scholars have studied identification processes by UAV remote sensing in vegetation fields. However, this vast body of research and applications still needs to be synthesized further to provide guidance and reference for the further application of UAV remote sensing technology in grasslands, offering more possibilities for the precise identification and management of grassland vegetation. Meta-analysis reviews contribute to reviewing the scope, sources, geographical distribution, and temporal patterns of research on the subject, as well as the effects of various factors on identification accuracy, such as spatial resolution, sensor types, and methods. In recent years, several scholars have already provided reviews concerning remote sensing-based applications in the agriculture field, such as agro-environmental monitoring based on UAV imagery (Eskandari et al., 2020), land use mapping and monitoring (Joshi et al., 2016), estimate aboveground biomass of grasslands (Morais et al., 2021), wetland classification (Mahdianpari et al., 2020a) and wetland monitoring (Adeli et al., 2020). Hence, this study offers a meta-analysis and review of vegetation identification using UAV remote sensing, providing valuable insights for fellow researchers in the field.

Therefore, this article aimed to review the application of unmanned aerial vehicle (UAV) remote sensing data in the identification field and conduct a meta-analysis of relevant literature in the past decade. It illustrates the importance of UAV agricultural identification research. It reviews the development trends in UAV agricultural identification research, aiming to facilitate its better and faster application in farm management and ecological conservation, thereby promoting its better and quicker application in agricultural management and environmental conservation. The second section briefly overviews the mainstream unsupervised and supervised classifiers. The third section introduces the method of meta-analysis. The results and discussions of the meta-analysis are elaborated in Section 4. In Section 5, we reviewed in detail the research progress and future development opinions on the application of UAV remote sensing in the vegetation identification process. In the final section, we summarized the conclusions.

## 2 Overview of remote sensing vegetation classifier

**Figure 1:**
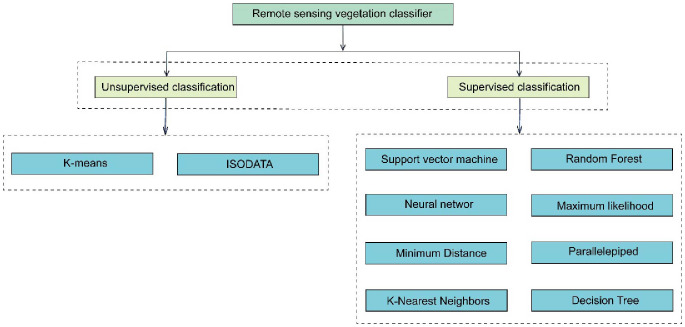
Remote sensing vegetation classifier.

Recently, vegetation classification methods based on remote sensing technology have significantly progressed. Studies have shown more accurate vegetation classification through the continuous development of feature extraction, classifier selection, and training. Up to now, many classical machine learning algorithms can effectively distinguish different vegetation types according to the extracted features, which provides strong support for the application of remote sensing technology. The remaining part of this section focuses on several commonly used vegetation classifiers in remote sensing, mainly including unsupervised and supervised classification (**Figure 1**).

### 2.1 Unsupervised classification

Unsupervised classification categorizes pixels or objects without prior knowledge or training samples (Behmann et al., 2014; Puletti et al., 2017). Using statistical algorithms like clustering or self-organizing maps can identifies patterns and similarities in remote sensing data, revealing natural clusters corresponding to different land cover or vegetation types.

#### 2.1.1 K-means

K-means, a foundational unsupervised clustering algorithm introduced in the 1960s, is pivotal in remote sensing image classification and agricultural monitoring. For instance, Gao et al (Gao and Li, 2017). used K-means to map degraded grasslands in the Qinghai-Tibet Plateau. However, it’s sensitive to noise and outliers, and finding the best number of clusters (K) is essential. With the deepening of algorithm research, researchers have proposed various improvement methods, such as K-means++, to enhance the selection of initial cluster centers, reduce convergence time, and improve classification accuracy (Yuchechen et al., 2020). As demand for processing large-scale remote sensing data grows, research into distributed computing and parallelization of K-means is increasingly essential.

#### 2.1.2 Iterative self-organizing data analysis technique

ISODATA emerged in the 1970s as an enhancement to basic clustering algorithms like K - means (Hemalatha and Anouncia, 2017). It adjusts cluster centroids and data assignments iteratively using similarity metrics, but with a dynamic approach to determining cluster numbers through split or merge operations. This adaptability makes ISODATA helpful in handling complex datasets, yet it’s sensitive to noise and outliers and may produce overlapping clusters. Currently, ISODATA classification is widely used in agricultural remote sensing image analysis. multi-dimensional features like spectral bands and vegetation, it can effectively distinguish crop types can effectively distinguish crop types by leveraging multi-dimensional features like spectral bands and vegetation. This makes it valuable for monitoring crop distribution and land-use patterns and enhancing agricultural productivity. For example, R et al. used LandSat7 and LandSat8 imagery data for unsupervised image classification with ISODATA, categorizing images into land-use classes (roads, urban areas, vegetation, water bodies, fallow land, mining areas, and barren land) with an accuracy of 83% - 86% across all categories (R, 2020). Moreover, ISODATA can be combined with machine-learning algorithms for post-processing, such as using a support vector machine to refine ISODATA-generated clusters to potentially boost overall classification accuracy.

### 2.2 Supervised classification

Supervised classification, utilizing training samples with known class labels, is a technique for categorizing pixels or objects in remote sensing imagery (Han et al., 2022). This method enables precise class assignment to image features by leveraging prior knowledge and training samples, making it valuable for applications such as land cover mapping, environmental monitoring, and resource management (César Pereira Júnior et al., 2020).

#### 2.2.1 Support vector machine

SVM is a widely used classification method in remote sensing (Melgani and Bruzzone, 2004; Mountrakis et al., 2011), first proposed by Vapnik et al. in the 1970s (Shebl et al., 2022; Sheykhmousa et al., 2020). It is particularly effective for high-dimensional datasets and image classification tasks (Bazi and Melgani, 2006; Huang et al., 2018; Mazzoni et al., 2007), offering strong performance even with small training sets by maximizing the margin between classes (Kuo et al., 2014; Mathur and Foody, 2008a; Srivastava et al., 2012). SVM also handles noise and outliers well, addressing data quality variations common in remote sensing (Pal and Mather, 2006). Comparative studies show that SVM outperforms methods like maximum likelihood classifiers and avoids dimensionality issues such as the Hughes Effect (Oommen et al., 2008). Meanwhile, there are also studies showing that SVM models optimized by techniques such as Particle Swarm Optimization have further enhanced the classification accuracy (Jia et al., 2022). Despite the many advantages of SVM, some challenges cannot be ignored, such as the selection of appropriate parameters and the difficulties faced by many non-specialist users in interdisciplinary applications (Chunjuan et al., 2016; Shebl and Csámer, 2021). Future developments may involve integrating SVM with multi-view learning using diverse remote sensing data sources (Hao et al., 2022), and leveraging quantum computing to improve efficiency in processing large-scale datasets (Delilbasic et al., 2024).

#### 2.2.2 Random forest

Introduced by Leo Breiman in 2001, RF is a robust machine-learning method that aggregates numerous decision trees, each built from randomly selected subsets of the dataset and features (Naidoo et al., 2012; Richman and Wüthrich, 2020). t is widely used in remote sensing for tasks such as image classification and land cover extraction due to its ability to effectively handle high-dimensional data and large datasets (Belgiu and Drăguţ, 2016). It mitigates overfitting risks and enhances generalization through random feature and sample selection (Harris and Grunsky, 2015). The ensemble approach of RF boosts classification accuracy and improves interpretability and reliability by providing results from individual decision trees (Belgiu and Drăguţ, 2016). For instance, using high-resolution multispectral imagery, Bhatt and Maclean successfully applied RF to classify forest vegetation and adjacent wetland communities (Bhatt and Maclean, 2023). However, RF has some drawbacks, such as a lack of interpretability and performance sensitivity to parameter selection (Guo et al., 2022; Vogels et al., 2019). Future research may combine RF with deep neural networks to leverage the feature extraction capabilities of deep learning and the classification strengths of RF, potentially achieving more efficient vegetation classification (Petrovska et al., 2020).

#### 2.2.3 Neural network

NN has a long history, with the first simple neural network models developed in the 1940s (ROSENBLATT, 1958). However, these early models were limited in handling complex classification tasks. T The development of multi-layer perceptrons (MLPs) in the 1980s and 1990s significantly enhanced neural network performance (David et al., 1986; Sumsion et al., 2019). The advent of deep learning in the 2010s, particularly convolutional neural networks (CNNs) and recurrent neural networks (RNNs), further revolutionized vegetation classification (Li et al., 2019b; Verbeiren et al., 2008; Zhang et al., 2016; Ho Tong Minh et al., 2018; Isola et al., 2017). CNNs have been incredibly effective in extracting spatial features from remote sensing imagery (Krizhevsky et al., 2017). Gambarova et al. successfully applied artificial neural networks (ANNs) to classify rare vegetation communities (Gambarova et al., 2008). Papp et al. demonstrated superior accuracy (99.61%) using ANNs compared to SVM on high-resolution hyperspectral images (Papp et al., 2021). However, neural networks face challenges like initial weight selection and slow convergence, impacting classification outcomes.

#### 2.2.4 Maximum likelihood

The ML classifier has been a staple in remote sensing since the early days of digital image processing. Initially applied to simple datasets, it assumes spectral signatures follow a normal distribution. As remote sensing evolved and more complex datasets emerged, ML was modified to handle non-Gaussian distributions and multi-class classification, including mixture models for complex class distributions. ML effectively classifies land cover and vegetation, especially in agriculture, where it maps crop types, irrigation patterns, and assesses crop health. Its ability to process large datasets and perform well in complex landscapes makes it crucial for modern agricultural decision-making. For instance, Otukei et al. applied ML to LandSat imagery in Kibale County, Uganda, achieving over 87% accuracy in classifying land cover types such as forest, wetland, and pasture (Otukei and Blaschke, 2010). However, ML requires accurate estimates of class means and covariances, which can be challenging, particularly with limited or noisy training data. Improving parameter estimation or relaxing the normality assumption remains a key research focus.

#### 2.2.5 Minimum distance

The MD classifier, a simple and intuitive supervised classification method, calculates the distance between a pixel’s feature vector and the mean feature vectors of known classes to assign the pixel to the closest class. In agricultural remote sensing, it has been used for land cover classification, including crops and soil variations (Castillejo-González et al., 2009). However, the performance of the minimum distance classifier highly depends on the choice of distance metric. Different distance metrics may lead to different classification results, and finding the optimal metric for a specific vegetation classification task can be challenging.

#### 2.2.6 Parallelepiped

The parallelepiped algorithm classifies data by defining multidimensional thresholds for each class in the feature space using parallelepiped boxes. Its simplicity and versatility make it widely used in remote sensing tasks such as land cover mapping, environmental monitoring, and resource management (Jensen, 2013). Yadav et al. applied this method to UAV-captured multispectral images to identify volunteer cotton in cornfields (Yadav et al., 2019). However, the technique assumes rectangular class distributions in the feature space, which may not be accurate for many vegetation identification scenarios. Future developments should focus on handling outliers and adapting the parallelepiped shape to match actual class distributions better.

#### 2.2.7 K-nearest neighbors

The K-NN algorithm is a simple yet effective machine learning method for pattern recognition and classification. It classifies data points based on the majority class of their nearest neighbors in feature space, using distances such as Euclidean or Manhattan (César Pereira Júnior et al., 2020). The algorithm’s performance depends on the choice of k value and distance metric, and its non-parametric nature allows it to adapt to various data distributions. K-NN has been extensively used in agricultural remote sensing to classify crops and land cover types, leveraging spectral information from imagery to accurately categorize agricultural landscapes, including crop types and soil variations. Its simplicity and non-parametric nature make it suitable for handling complex, multi-dimensional feature spaces. For instance, Verma et al. applied object-based K-NN techniques to classify land cover in a 5000-hectare grazing property in Australia, achieving overall classification accuracy of 86% (Verma et al., 2014). However, K-NN’s computational cost can be substantial for large datasets, as it involves calculating distances between each query pixel and all training samples.

#### 2.2.8 Decision tree

The DT classification algorithm is fundamental in machine learning and is widely used for predictive modeling and pattern recognition. It constructs a tree-like structure where nodes represent features, and leaf nodes represent class labels (Pal and Mather, 2003). DT recursively partitions the feature space to minimize impurity and enhance class homogeneity, offering interpretability and operational efficiency. Verma et al. applied DT to classify cultivated and non-cultivated areas and crops using satellite images (Verma et al., 2016). Integrating texture features with vegetation indices achieved an 89.42% accuracy in classifying Indian satellite IRS-P6, LISS IV remote sensing images. However, DT is prone to overfitting, especially with noisy or high - dimensional data. Extension and ensemble methods like RF and Gradient Boosting have been developed to address this while retaining interpretability. Further research is needed to enhance DT’s prediction accuracy and expand its application scope through continuous refinement.

Although the recent vegetation identification methods based on UAV remote sensing have made much progress, there are still many challenges. For unsupervised methods, determining the optimal number of clusters and mitigating the impact of initial conditions on the clustering outcome remain significant hurdles. For supervised methods, particularly complex models like NN, the risk of overfitting and the reliance on extensive, high-quality training datasets pose substantial challenges. Additionally, the computational demands of advanced machine learning algorithms necessitate efficient processing solutions, such as cloud computing, to handle large-scale UAV remote sensing data effectively. Therefore, we will explore the performance of different classification methods in different situations and the impact of various factors on classification performance through previous relevant studies provide effective programs for further research, especially the tradeoff between specific classification problems and method selection.

## 3 Meta-analysis methods

### 3.1 Literature collection

**Figure 2:**
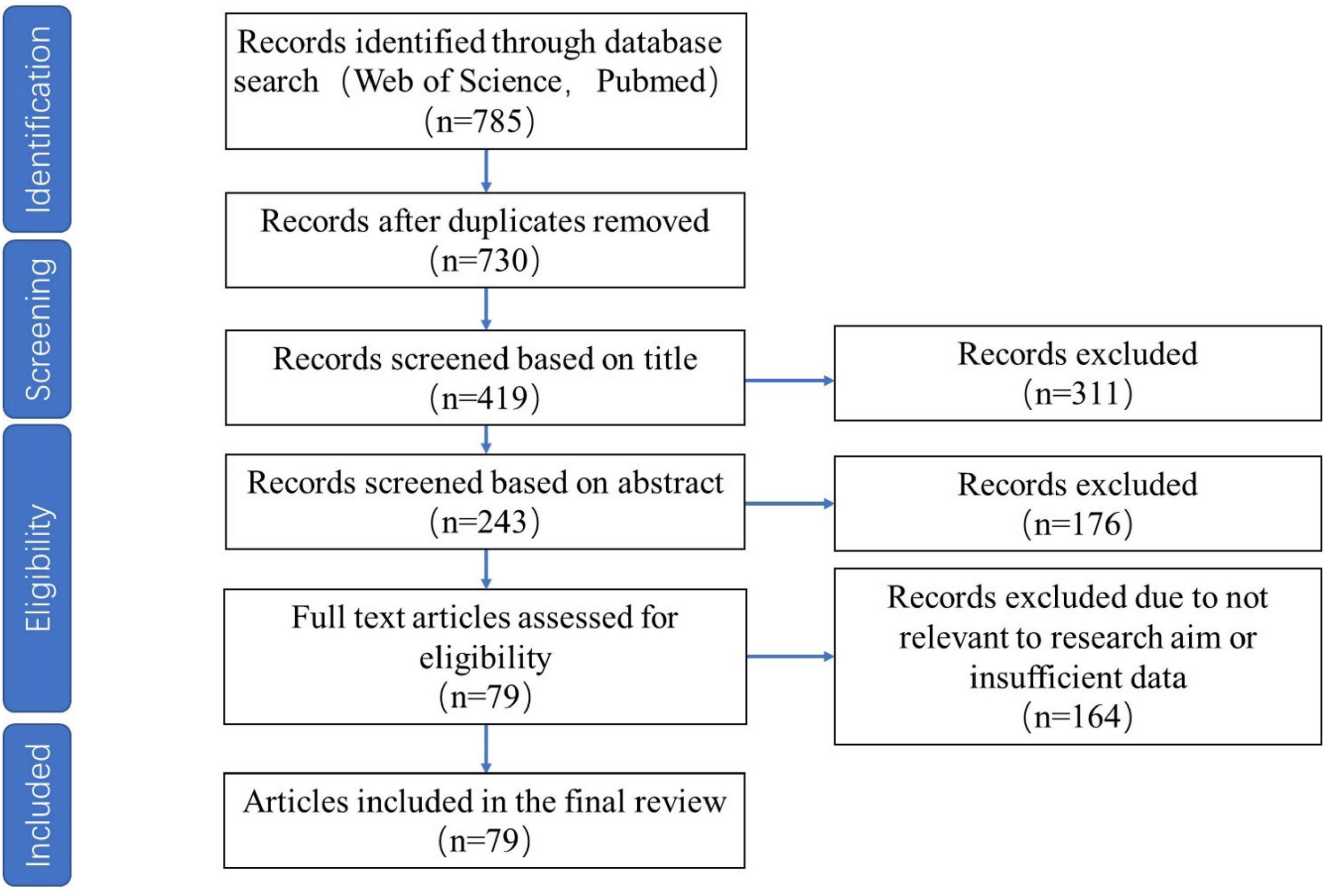
PRISMA flowchart demonstrating the selection of studies.

This review involved three steps: literature collection, filtering relevant papers, and comprehensive analysis and review of relevant studies. We performed a search of the “Web of Science (WoS)” and “PubMed” databases in December 2022 using the designed search query to identify relevant literature for this comprehensive review. In our meta-analysis, we specifically focused on the application of unmanned aerial vehicle (UAV) remote sensing for identifying smaller-scale vegetation types, such as crops, herbaceous plants, and fruits, while excluding studies related to woody plants. By narrowing our scope to non-forest vegetation, we aim to provide a more detailed examination of the emerging applications, challenges, and limitations of UAV remote sensing technologies in scenarios involving less extensive and potentially more varied vegetation types, offering insights into their precision and versatility in diverse ecological contexts. The search was structured with variations on each key term included via the OR operator. To determine recent findings while keeping the workload at controllable levels, our search focused on articles within the ten years, from 2013 to 2022. The Preferred Reporting Items for Systematic Reviews and Meta-Analyses (PRISMA) were applied for study selection (Moher et al., 2009). This search strategy resulted in a total of 785 unique papers. Then, 730 articles were obtained after removing duplicates. Next, we filtered the initial result by document title and abstract, reducing the number of results to 243. After investigating the 243 papers in detail and after eligibility assessment, 79 were selected to collect data for further meta-analysis, as shown below. **Figure 2** depicts the selection of relevant papers based on the PRISMA flow diagram. It must also be noted that studies related to f forests were excluded from this study and, as a result, are not captured in this search. The following norms were also applied to select relevant papers:

1. Articles included a quantitative accuracy assessment that reported overall accuracy (OA) of classification or identification.
2. Only research papers published in journals were included. This meta-analysis does not include conference papers due to their lack of information.
3. Articles were limited to study with UAV-based data as the sole or primary source.

### 3.2 Data extraction

For the following comprehensive review, the parameters in **Table 1** were extracted by reviewing 79 publications in detail. The parameters include general literature characteristics fields such as title, author, year, and literature source, as well as specific fields related to identification, including fly altitude, sensor type, spatial resolution, and classifier. We defined a “case” as the unique combination of the features and corresponding result (overall accuracy), e.g., if a study used an identical data set but identified using different methods; then we treated them as other cases. Furthermore, if an article gives multiple filter sizes, patch sizes, window sizes, sizes of training data sets, and so on for the same input data, only the version with the highest overall accuracy was used in the meta-analysis. In addition, the test set is preferred for results with the same input data, followed by the validation set, and finally the training set in an article.

**Table 1:** Fourteen parameters used for Meta-analysis.

ID	Attribute	Type	Categories
1	Title	Text	
2	Authors	Text	
3	Year	Classes	
4	Literature source	Text	
5	Evaluation indices	Classes	Overall accuracy; user’s accuracy; producer’s accuracy; kappa coefficient; F1-Score; not available;
6	Study field	Text	
7	Fly altitude	Numeric	
8	Case study purpose	Text	
9	Sensor type	Classes	Hyperspectral; multispectral; RGB; SAR; LIDAR; others
10	Spatial resolution	Numeric	
11	Number of classes	Numeric	
12	Classifier	Classes	SVM; RF; CNN; DT; MLC; ISODATA; ANN; Others
13	Enhancement methods for the input data	Classes	Texture; ancillary data; spectral indices; without
14	Overall accuracy	Numeric	

### 3.3 Selected references

**Figure 3:**
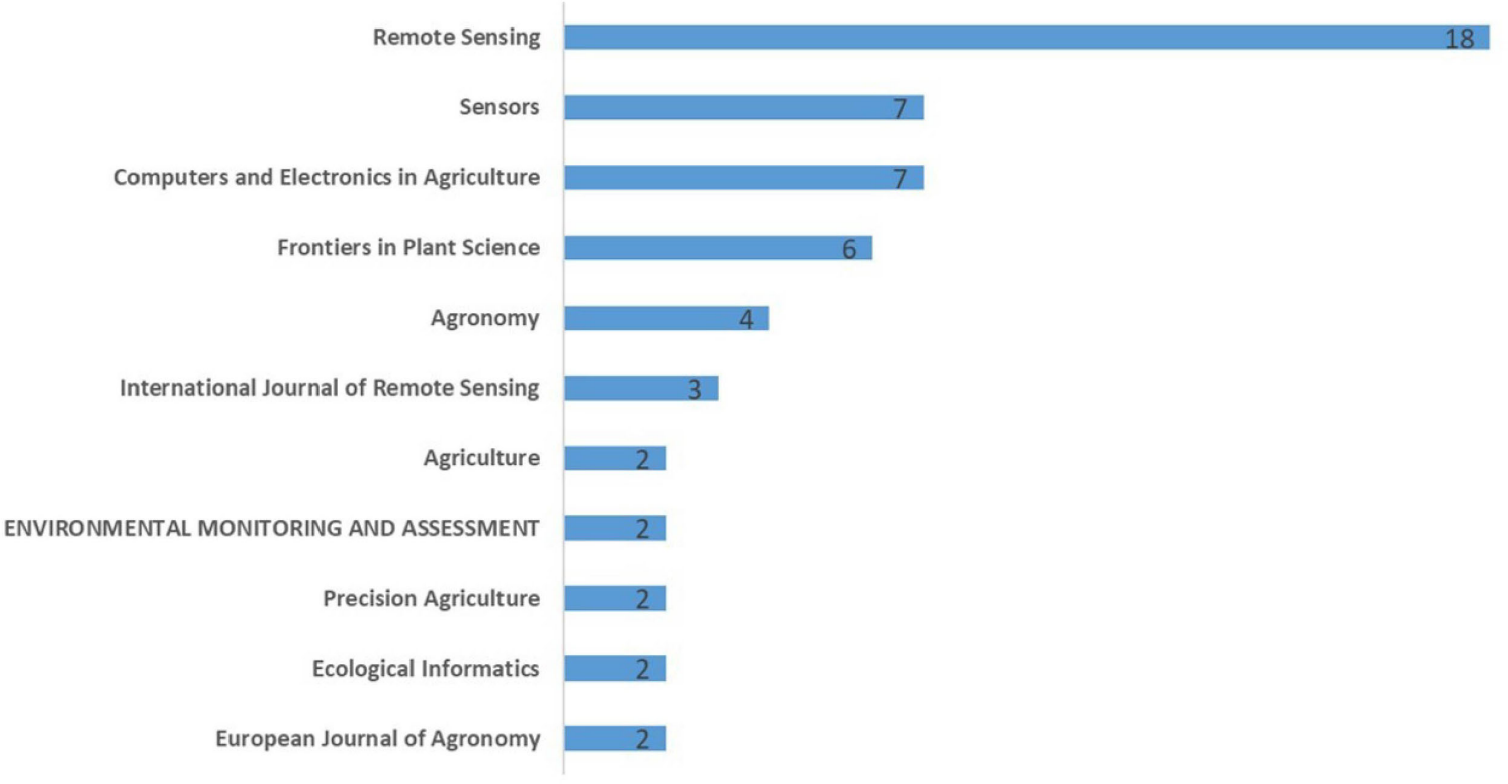
Number of primary literature sources.

For the selected 79 journal papers, most articles were published in the 11 journals listed in **Figure 3**. The remaining journals involving only one paper are not listed. As can be seen from the figure, from the perspective of the number of published articles, the top five journals with the number of published articles, in turn, are: Remote Sensing (23%), Sensors (9%), Computers and Electronics in Agriculture (9%), Frontiers in Plant Science (8%) and Agronomy (5%). Notably, they accounted for 53% of all the selected papers, more than half. Among them, the number of Remote Sensing journals is the largest, with 18 articles.

### 3.4 Performance evaluation index

**Figure 4:**
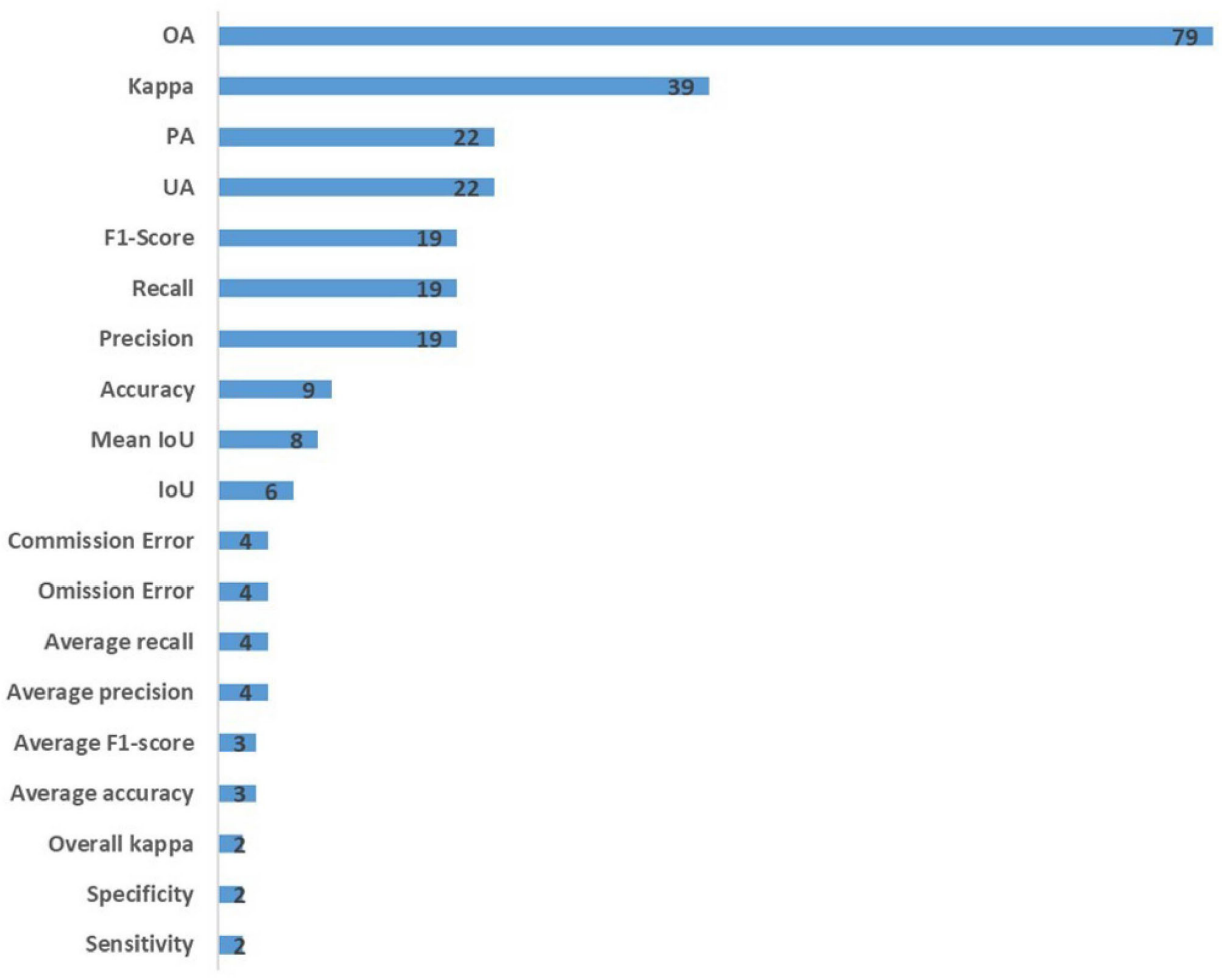
Overview of evaluation indexes used to evaluate the results of studies.

**Figure 4** shows the overall situation of evaluation indexes used to evaluate the results of studies. A majority of studies use multiple metrics to assess the identification performance. In addition to the overall accuracy, the Kappa coefficient, producer accuracy (PA), and user accuracy are the indicators most commonly used in 39, 22, and 22 studies, respectively.

## 4 Results and discussion

### 4.1 General characteristics of studies

**Figure 5:**
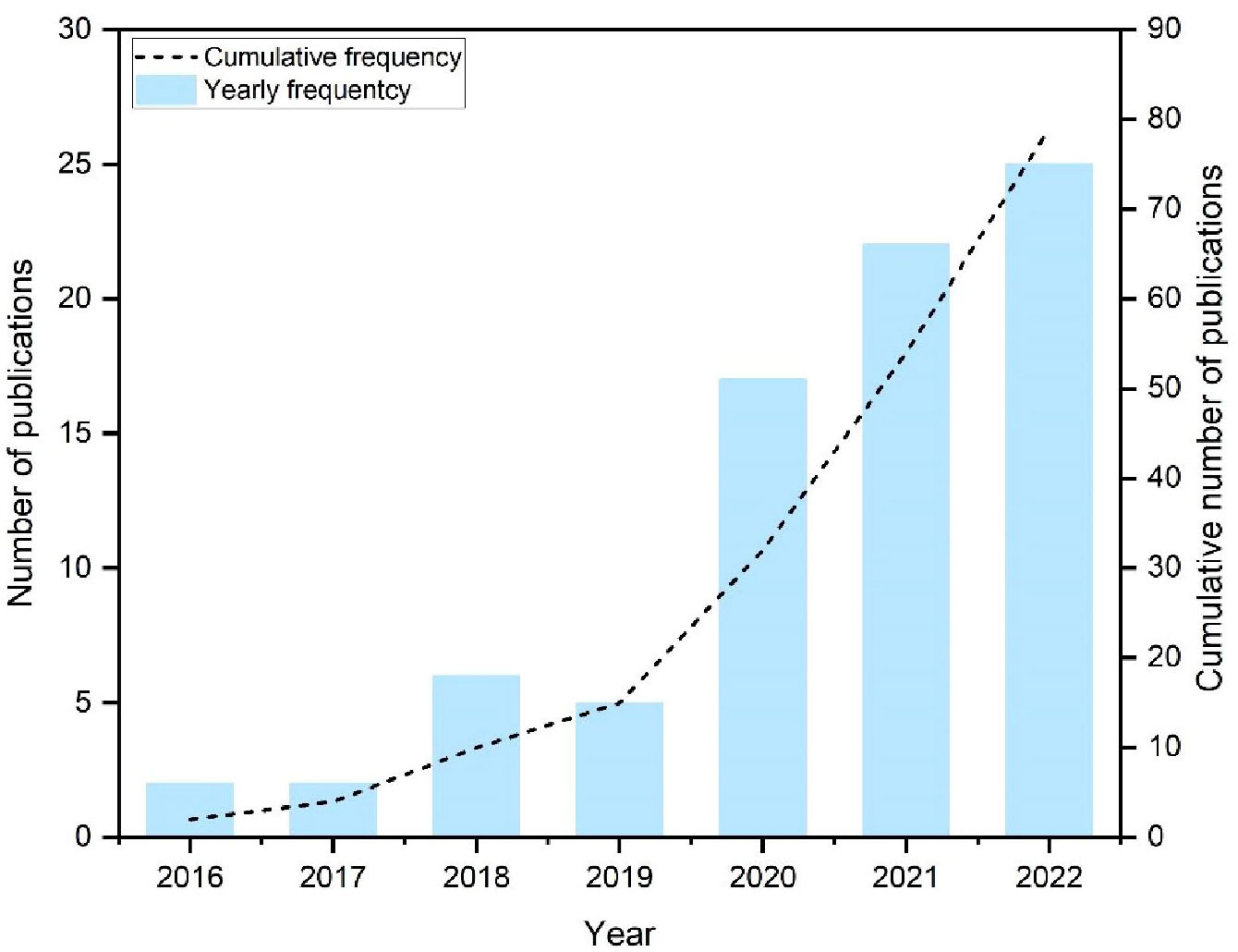
Cumulative and yearly frequency of relevant publications.

As shown in **Figure 5**, the number of identification studies based on UAV remote sensing has increased significantly over the past decade, indicating an increasing interest in research in this area. Simultaneously, the rate of increase also showed an upward trend. This illustrates that the employment of UAV data gained the researchers’ attention. The number of published articles is expected to expand in the coming years.

**Figure 6:**
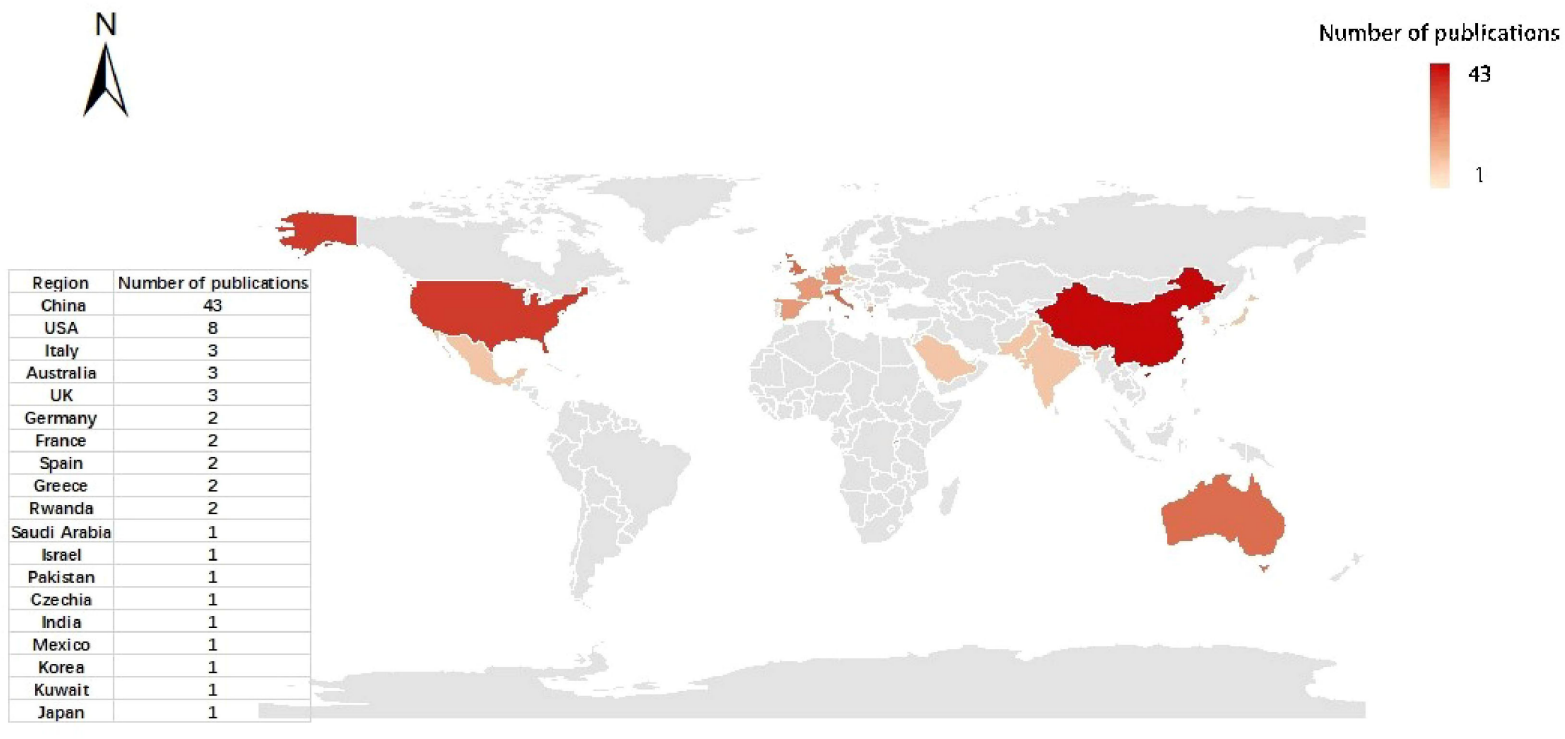
Spatial distribution of relevant publications. The degree of color is proportional to the number of studies.

The number of articles by country is uneven (**Figure 6**), and the depth of color is proportional to the number of studies. We mapped study sites from the 79 papers and found that applications of UAV data have been widely distributed in research worldwide, mainly in Asia, Europe, and North America. And they accounted for 63%, 19%, and 11% of all studies collected, respectively. Most studies have been carried out in Asia, notably in China 43 studies. Countries with more than five studies are China (43) and the United States (8). Italy, Australia, and the UK followed it. To sum up, the studies for our meta-analysis covered a broad scope of geographical areas and abundant topics.

### 4.2 Parameters and studies performance

#### 4.2.1 Case study target

**Figure 7:**
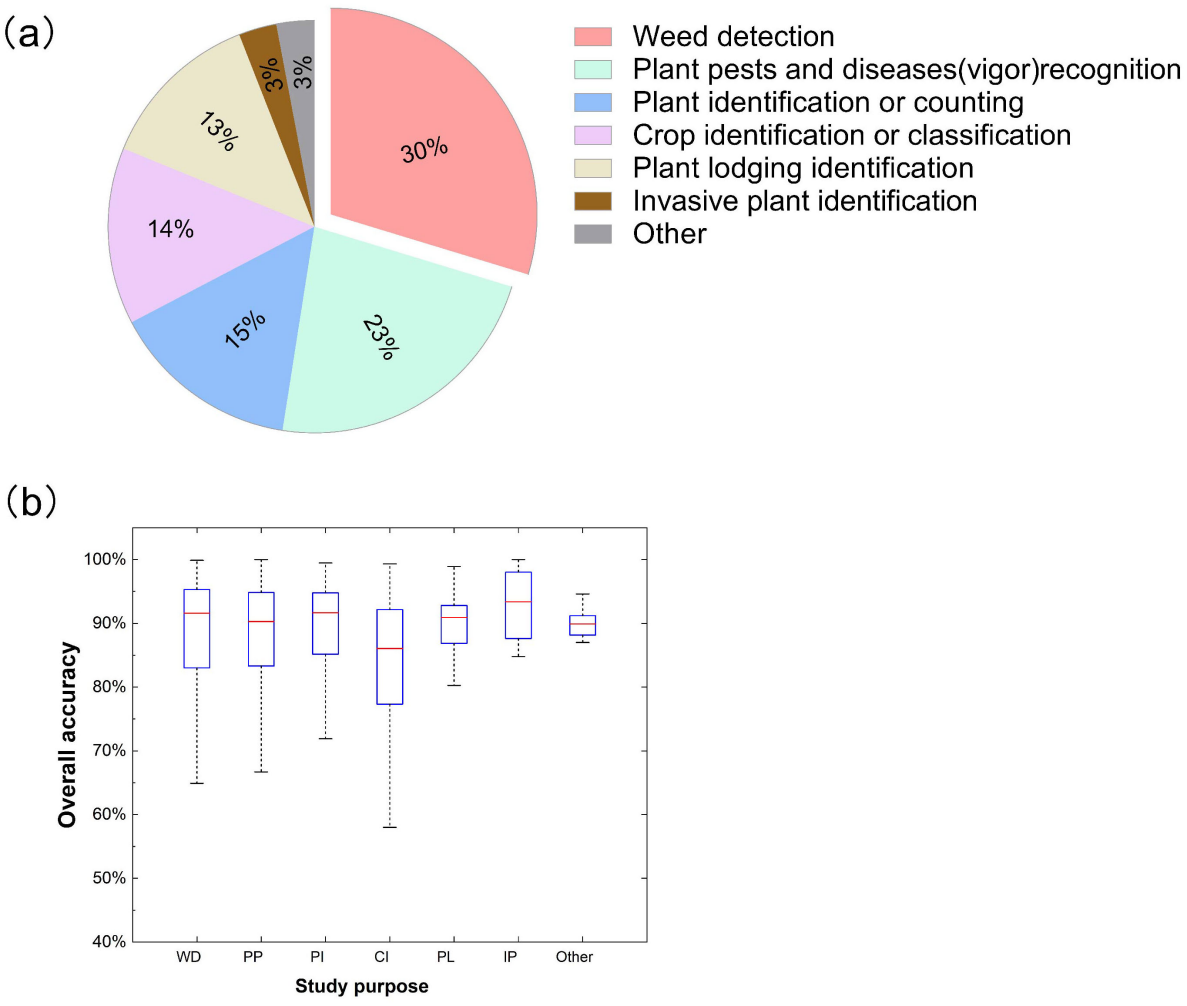
Distribution **(a)** and overall accuracy (OA) **(b)** for different study targets in related publications. WD, weed detection; PP, plant pests and diseases (vigor) recognition; PI, plant identification or counting; CI, crop identification or classification; PL, plant lodging identification; IP, invasive plant identification.

Most studies focused on weed detection, plant pests and diseases (vigor) recognition, and plant identification or counting (**Figure 7a**) hey accounted for 30%, 23%, and 15% of all studies. This demonstrates that, with the wide application of remote sensing in various fields of agriculture, weed identification has essential and diverse application prospects in agricultural remote sensing (dos Santos Ferreira et al., 2017; LÓPez-Granados, 2010). In the weed identification scenario, when weeds and crops are intermingled within the same image, the issue becomes more complex due to their similar spectral characteristics (Weiss et al., 2020). UAV remote sensing paves the way for exploring new avenues in precision vegetation classification (Lu and He, 2017). de Camargo et al. (2021) proposed optimizing a deep residual convolutional neural network (ResNet-18) for classifying weed and crop plants in UAV imagery. Rozenberg et al. (2021) used a simple unmanned aerial vehicle (UAV) to survey 11 commercial plots of Allium cepa L. They used ML and SVM to study their late weed classification and spatial patterns, which constitute an essential step in developing accurate weed control management in onion fields. Timely and precise weed identification would enhance the understanding of weed growth patterns and spatial distribution patterns for analyzing and forecasting the mechanism of growth and reproduction and provide further convenience for practitioners and researchers to manage weeds in farmland, thereby increasing crop productivity (Sulaiman et al., 2022; Zhang et al., 2023b). The availability of UAV data in recent years has made weed detection possible with less time and effort (Alexandridis et al., 2017; Stroppiana et al., 2018). After mastering the growth and distribution of weeds, targeted herbicide spraying can effectively avoid the problems of herbicide waste and agricultural ecological environment pollution.

Simultaneously, the recognition of plant pests and diseases and identifying plant vitality have increasingly become a hot issue based on remote sensing (Abd El-Ghany et al., 2020; Mahlein, 2016; Mahlein et al., 2012). Some researchers (Khan et al., 2021a) used hyperspectral imaging technology to identify wheat powdery mildew disease without apparent symptoms and estimate the corresponding disease severity. This study used remote sensing to detect and prevent crop diseases early, which is more efficient than traditional methods relying on disease symptoms. Wang et al. (2020) investigated the use of UAV to collect high-resolution remote sensing images of cotton fields in three known cotton root rot (CRR)-infested areas of Texas, classifying CRR-infected and healthy plants in cotton fields. Therefore, there is no doubt that the rapid monitoring of the vegetation health status and the prediction of early plant diseases by UAV is becoming an active research field, which has also made a significant contribution to crop yield and farmers’ income (Neupane and Baysal-Gurel, 2021). Our meta-analysis also demonstrated this point. Food security is one of the hot issues in today’s society and may be one of the reasons for this situation (Lu et al., 2022).

It is worth noting that, among the research we have collected, studies related to crop classification only account for about 14%. Several reasons for this can be hypothesized: (1) farmland ground sample acquisition is time-consuming and labor-intensive, and data storage is scattered and discontinuous (Ma et al., 2021); or (2) related technologies need to be further improved, and some traditional machine learning methods cannot meet the generalization requirements of increasingly complex farmland crop classification. Compared to industries like forestry, the application of drone remote sensing in crops, especially grassland, classification lags and requires further research investment. The complexity of various grass plants on the grassland may hinder the acquisition and differentiation of ground reference data (Dong et al., 2019). Additionally, it increases the likelihood of different spectra for the same objects, thereby complicating the identification of plant individuals and species. In early grassland vegetation classification mapping, visual interpretation or the delineation of broad grassland categories was predominantly used (Li et al., 2005). In recent years, with the advancement of UAV remote sensing technology and enhancements in spectral resolution, the precise identification of specific grass species has emerged as a new trend in grassland vegetation classification mapping (Melville et al., 2019). Given the smaller research objects within grass vegetation, higher sensor accuracy and data acquisition requirements are essential. Algorithms used in other fields may require enhancements or redevelopment before they can effectively apply to grassland research. It is worth noting that about 6% of the study is relatively small and belongs to other specialized research, such as invasive plant identification (Qian et al., 2020) and cultivated land identification (Lu et al., 2017).

**Figure 7b** shows a box plot graph of overall accuracy (OA) regarding the types of case study purpose, including the accuracy of weed detection, plant pests and diseases (vigor) recognition, plant identification or counting, crop identification or classification, plant lodging identification, invasive plant identification and other. Invasive plant identification had the highest median identification accuracy. However, these reference values need further study and validation due to the small sample size of invasive plant identification and others (less than 5%). In addition, plant identification or counting had the highest median accuracy (∼91.67%), and weed detection had the second-highest median accuracy (∼90.60%), with a difference of 0.07%. Among them, plant identification or counting was superior to weed detection in terms of variation in overall accuracies. Nevertheless, crop identification or classification had the lowest median accuracy (∼86.05%), and the lower and upper whiskers extended from 58.00% to 99.33%. Therefore, it is necessary to further develop and explore the application of crop classification in UAV remote sensing (Bouguettaya et al., 2022).

#### 4.2.2 Sensor types

**Figure 8:**
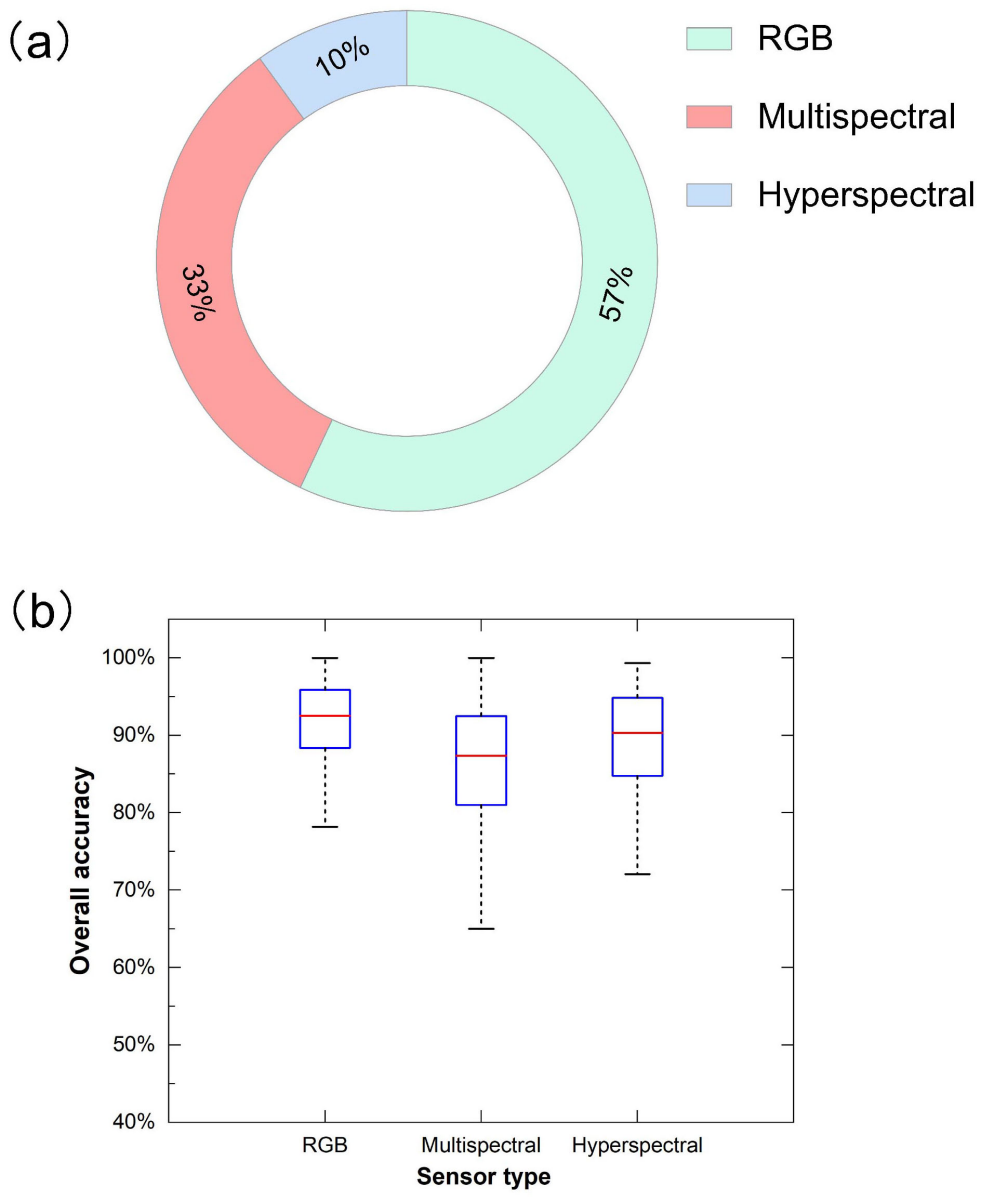
Distribution **(a)** and overall accuracy (OA) **(b)** for different sensor types in related publications.

Regarding sensor type, RGB sensors (visible sensors) are the most adopted type, with about 57%. The result is consistent with our assumption (Diez et al., 2021; Liu et al., 2021a). This shows that the RGB sensor has been the most commonly used within UAV remote sensing for identification studies, which benefits from its ease of installation for UAV platforms (López-García et al., 2022). Although RGB spectral data contains less spectral information, it has gradually become a shared data source for uncrewed aerial vehicle (UAV) plant species identification due to its low cost, easy acquisition, fast data processing speed, and high spatial resolution (Zheng et al., 2022). Followed by multispectral imaging at about 33% and hyperspectral imaging at about 10% (**Figure 8a**). The relatively low usage frequency of multispectral and hyperspectral data is not surprising, given their higher economic costs, especially for hyperspectral sensors (Adão et al., 2017). Their spatial resolution is also relatively lower than RGB (Kattenborn et al., 2021). One major obstacle in vegetation classification using UAV remote sensing has been the limited availability of fine spatial resolution data, insufficient spectral information, and insufficient processing capacity (Weiss et al., 2020). Multi-source remote sensing data can synthesize the advantages of each sensor; for example, the image after the fusion of RGB image and multi-spectral image has the characteristics of high spatial resolution and spectral resolution so that more abundant and accurate information can be extracted (Ashraf et al., 2012). Therefore, the fusion technology of UAV remote sensing images is increasingly becoming the preferred choice for effectively mitigating sensor limitations (Jabari et al., 2017). The fusion of UAV remote sensing images can be considered an image super-resolution problem, where the high-resolution source image assists in enhancing the low-resolution source image (Ma et al., 2019).

In addition to the widely-studied RGB, multispectral, and hyperspectral sensors, other types of sensors also hold potential in UAV-based vegetation identification (Tunca et al., 2023). Thermal infrared sensors, for instance, can detect the temperature differences of vegetation surfaces (Stutsel et al., 2021). Since plant water stress, pest infestations, and disease infections can all lead to changes in the plant’s surface temperature, thermal infrared sensors can provide unique insights into the physiological status of plants (Sagan et al., 2019).

At the same time, although LiDAR sensors are not as widely used as RGB, multispectral, and hyperspectral sensors in agricultural remote sensing, especially in vegetation classification and identification, they offer unique advantages by capturing the vertical structure of vegetation (Yel and Tunc Gormus, 2023). LiDAR can accurately measure vegetation height, canopy density, and biomass by generating three-dimensional point clouds, providing rich three-dimensional information for vegetation classification (de Conto et al., 2024). For example, LiDAR can effectively distinguish between healthy and damaged vegetation in crop areas, identify the spatial distribution and growth patterns of vegetation, thus improving the classification accuracy (Farhan et al., 2024). Moreover, LiDAR can provide detailed canopy structure information, which helps distinguish plant species that have similar spectral characteristics but differ in structure. Although LiDAR equipment is expensive and data processing is complex, brings its high-precision three-dimensional information to agricultural remote sensing, especially in vegetation classification under complex environments, demonstrates excellent potential (Karim et al., 2024). Future research should address the challenges related to cost and data processing to further promote the application of LiDAR in vegetation classification and identification.

The overall accuracy assessment shows the maximum median accuracy and the lowest variability for the RGB sensor, with almost 92.54%, followed by a hyperspectral sensor, with about 90.32%, and the multispectral sensor, with about 87.37% (**Figure 8b**). The underlying reason may be that UAV data is closer to the ground than satellite remote sensing data, and its spatial resolution advantage may be greater than the spectral resolution in recognition research. At the same time, we observed that some studies in the database extracted the texture features of remote sensing data to enhance its spatial features, which may be regarded as a further supplement to the spatial resolution (Liu et al., 2020; Stroppiana et al., 2018; Xiao et al., 2021). The multispectral sensor exhibits the highest maximum, yet its wider distribution and the lowest minimum show less consistent results than RGB and hyperspectral sensors. In addition, it’s worth noting that the median overall accuracy of the three sensor types surpassed 87%. The results show that the RGB sensor has the highest overall accuracy and muscular stability of the three sensor types, consistent with the most significant number of studies in **Figure 8a**).

#### 4.2.3 Spatial resolution

**Figure 9:**
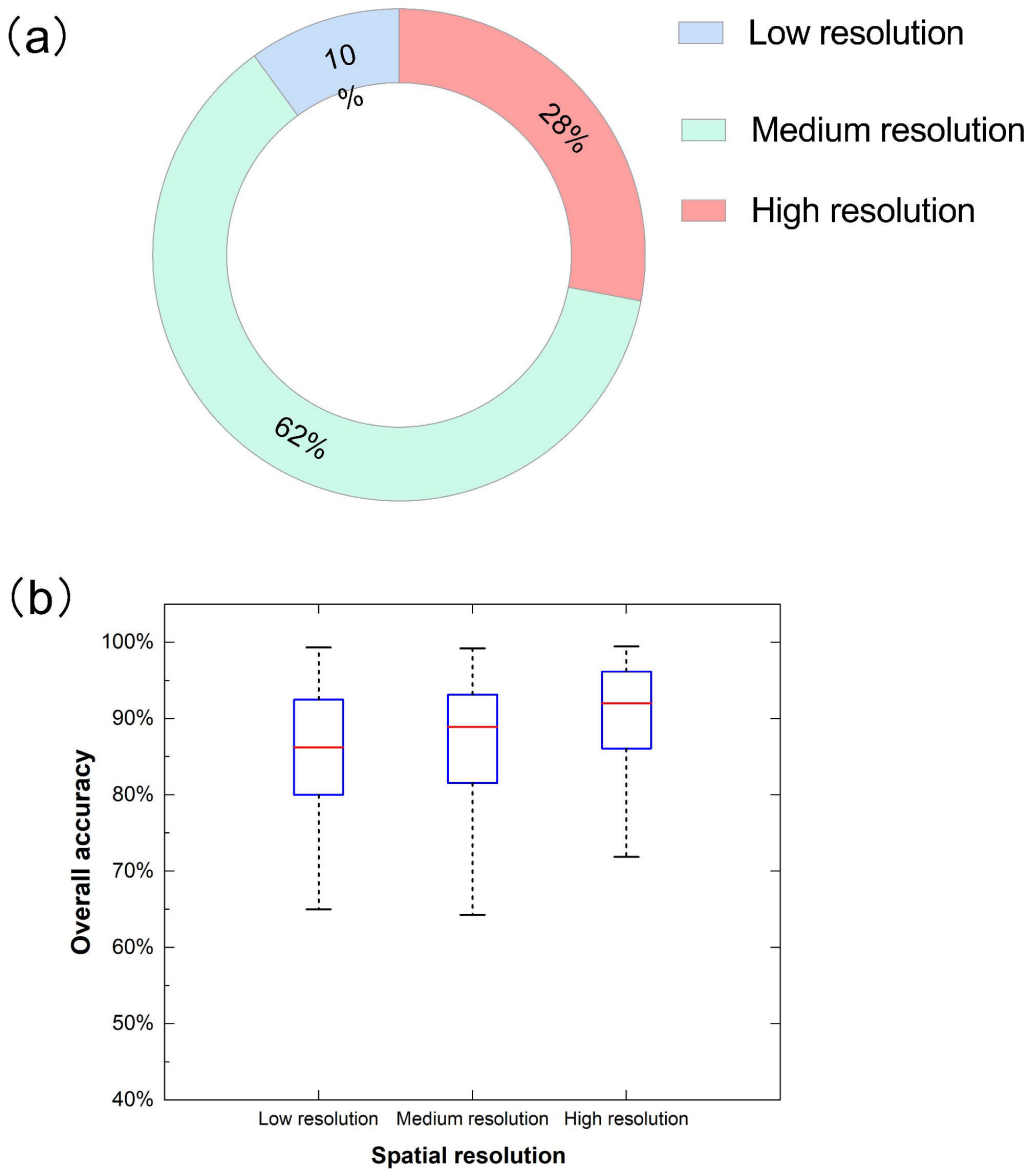
Distribution **(a)** and overall accuracy (OA) **(b)** for different spatial resolution in related publications.

Spatial resolution, the size of the smallest unit that can be distinguished in detail on a remote sensing image, is an indicator used to characterize the resolution of ground target details by an image. Medium spatial resolution UAV-based remote sensing imagery with a resolution between 1 and 10 cm is the most frequently used data source amongst those utilized for studies of agriculture identification with 62% (**Figure 9a**). This may be because these data contain rich spatial feature information and can also maintain the spectral resolution of UAV data at a relatively stable level. Furthermore, some researchers have proved that adding texture features improves recognition and classification accuracy under the same UAV multi-spectral data and spatial resolution (Sun et al., 2019; Tamouridou et al., 2016). They were followed by high resolution (between 0 to 1 cm) in approximately 28% of all studies. Regarding remote sensing imagery with low resolution (more than 10 cm), the case studies are most minor, with about 10%.

As the spatial resolution increases, so does its potential to achieve higher accuracy (**Figure 9b**). Simultaneously, the accuracy ranges from large to small in low-resolution, medium-resolution, and high-resolution images. This may be related to the number of their study samples. The maximum median overall accuracy is related to datasets with high spatial resolutions, with about 92.00% median accuracy, and the lower and upper whiskers extend from 71.90% to 99.47%. It has minimal variability because it gets a more compact shape than another shape, similar to the results of Tamiminia’s study (Tamiminia et al., 2020). However, it is imperative to recognize that the relationship between spatial resolution and accuracy is not unidirectional (Pampanoni et al. 2024). While higher spatial resolution offers the potential for enhanced performance, it also brings challenges. For instance, increased spatial detail can amplify the impact of noise present in the images (Silvestre et al. 2018). Sensor noise, atmospheric interference, or other sources of distortion become more pronounced at finer scales, potentially degrading the quality of the data and leading to misclassification. Additionally, in machine learning-based identification models, high-spatial-resolution data may contain an abundance of features, increasing the risk of overfitting (Zhang et al. 2012). Overfitted models perform well on the training data but fail to generalize effectively to new, unseen data, resulting in decreased accuracy during practical applications. Therefore, careful consideration must be given to data preprocessing, model selection, and parameter tuning when working with high-spatial-resolution images to mitigate these potential issues and fully harness the benefits of enhanced spatial detail.

**Figure 10:**
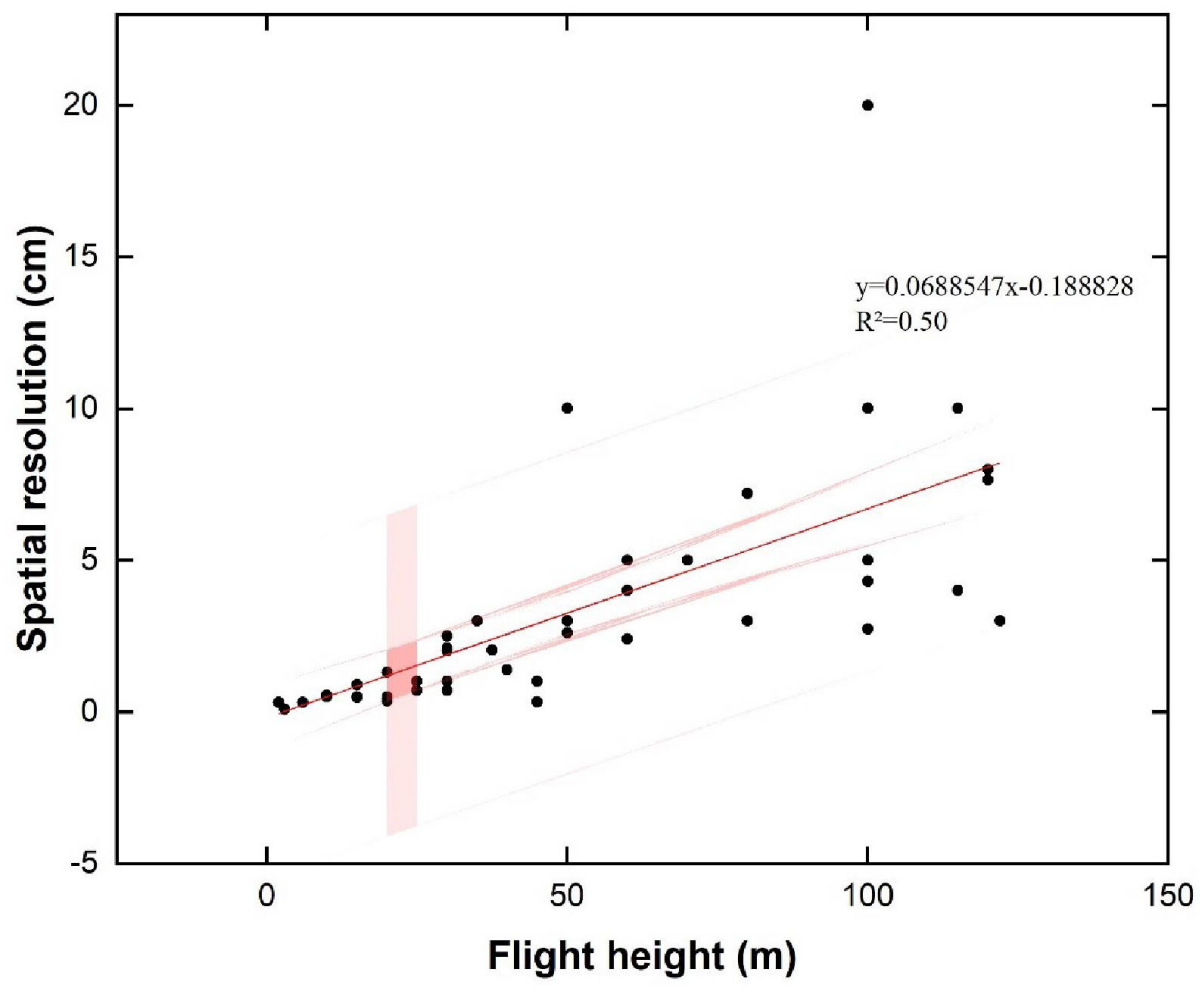
Relationship between flight altitude and spatial resolution based on relevant publications.

In addition, we also made a correlation fitting between UAV flight height and spatial resolution in related research (**Figure 10**). Notably, studies that did not report flight altitude, spatial resolution, or both were excluded from the analysis. At the same time, separate outliers outside the range of most values are not shown in the figure. In our study, we observed a positive correlation between the drone’s flight altitude and the spatial resolution of the captured images. Increasing flight height improves ground sampling distance (GSD) and reduces the spatial image resolution, thus increasing the coverage area. Therefore, in specific tasks under different environmental conditions, determining UAV flight height requires more flight tests to cover the appropriate research area and meet the experimental requirements. Additionally, as pointed out in the related review, spatial resolution is also associated with some other factors (such as the capture time of the sensor) (Eskandari et al., 2020), which is also worthy of our further study.

#### 4.2.4 Integrated application of sensor type and spatial resolution

In UAV-based vegetation identification, the selection of sensor type and spatial resolution significantly influences the accuracy and reliability of the results (Ubben et al., 2024; Xie et al., 2008). The sensor type determines the range and quality of spectral information captured, while spatial resolution affects the ground coverage of each pixel (Ubben et al., 2024). High-resolution sensors provide detailed information about vegetation structure but may also increase data processing complexity. Conversely, low-resolution sensors can cover larger areas but may fail to capture subtle vegetation features (Miao et al., 2024).

Different vegetation types exhibit unique physical and spectral characteristics, influencing the sensor requirements and spatial resolution needed for classification tasks (Pan et al., 2023; Rowan et al., 2021). For instance, crop identification often demands high spatial resolution RGB sensors to detect individual plants’ growth and health indicators (Khan et al., 2021b; Koh et al., 2021). Moreover, for grassland identification, multispectral sensors with moderate spatial resolution are typically essential (Al-Ali et al., 2020). These sensors enable the differentiation of various grass species and facilitate monitoring grassland coverage (Alexandridis et al., 2017).

Identification purpose also plays a key role in sensor selection and resolution determination. To identify early signs of vegetation pests and diseases, sensors must pick up subtle physiological and morphological changes (Zhang et al., 2023a, 2019). In such cases, multispectral or hyperspectral sensors with medium-to-high spatial resolution may be more appropriate (Ishengoma et al., 2021; Xiao et al., 2021).

Furthermore, the required spatial resolution for classification varies depending on the vegetation’s scale and complexity (Liu et al., 2025; Räsänen and Virtanen, 2019). For dense vegetation or fine-scale features, higher resolution may be necessary to distinguish individual plants or specific traits (Feng et al., 2015). On the other hand, broader vegetation types or more significant areas may require lower resolution to maintain processing efficiency without sacrificing classification accuracy (Peng et al., 2024; Zhang et al., 2024).

In conclusion, in UAV-based vegetation classification, achieving high accuracy is crucial but must be balanced with cost, mission objectives, and data processing needs. High-resolution sensors, while enhancing accuracy by capturing detailed spectral and spatial information, often entail higher costs, longer processing times, and more significant data storage requirements. Therefore, selecting the optimal sensor and resolution involves weighing the trade-offs between accuracy and practical constraints (Saltiel et al., 2022). Ultimately, the choice should align with the project’s objectives, ensuring an efficient balance between precision and operational feasibility (Ishida et al., 2018).

#### 4.2.5 Number of target categories

**Figure 11:**
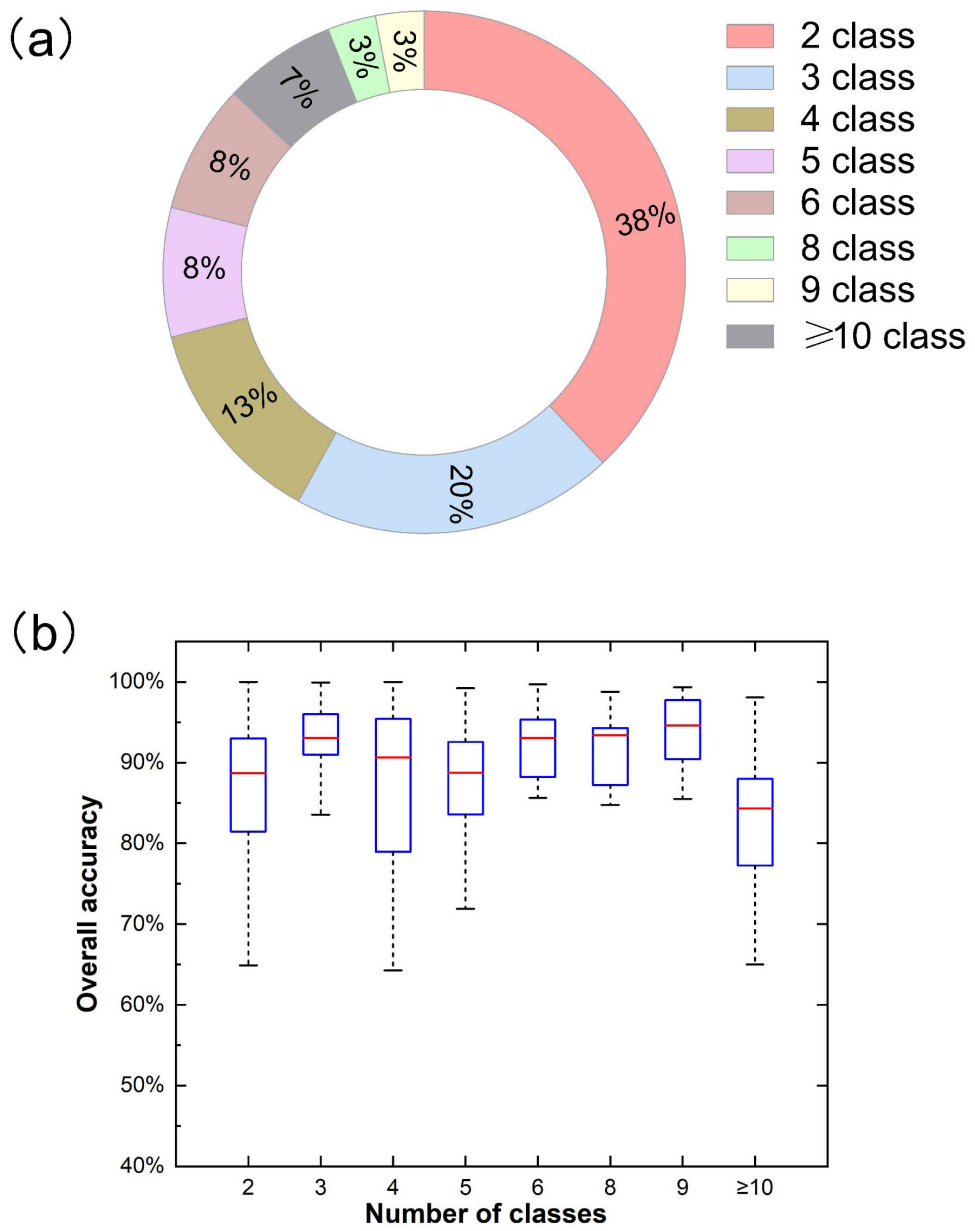
Distribution **(a)** and overall accuracy (OA) **(b)** for different number of classes in related publications.

Through the analysis of the number distribution of different target categories in related research, most studies identified a smaller number of target categories, such as two classes with almost 38%, three courses with nearly 20%, and four classes with about 13%, which their total has reached 71% (**Figure 11a**). In summary, with the increase in the number of target categories, the proportion of the number of studies is gradually decreasing.

As shown in **Figure 11b**), when the number of classes is 9, the median overall accuracy is the highest, at about 94.60%. This may be due to the small number of research samples, which is only 3%. In addition, when the number of classes is 3, the overall accuracy median is the highest, about 93.04%. Meanwhile, its interquartile range (IQR) and overall accuracy change less than other categories. This shows no clear relationship between number of classes and overall accuracy.

#### 4.2.6 Classification methods

**Figure 12:**
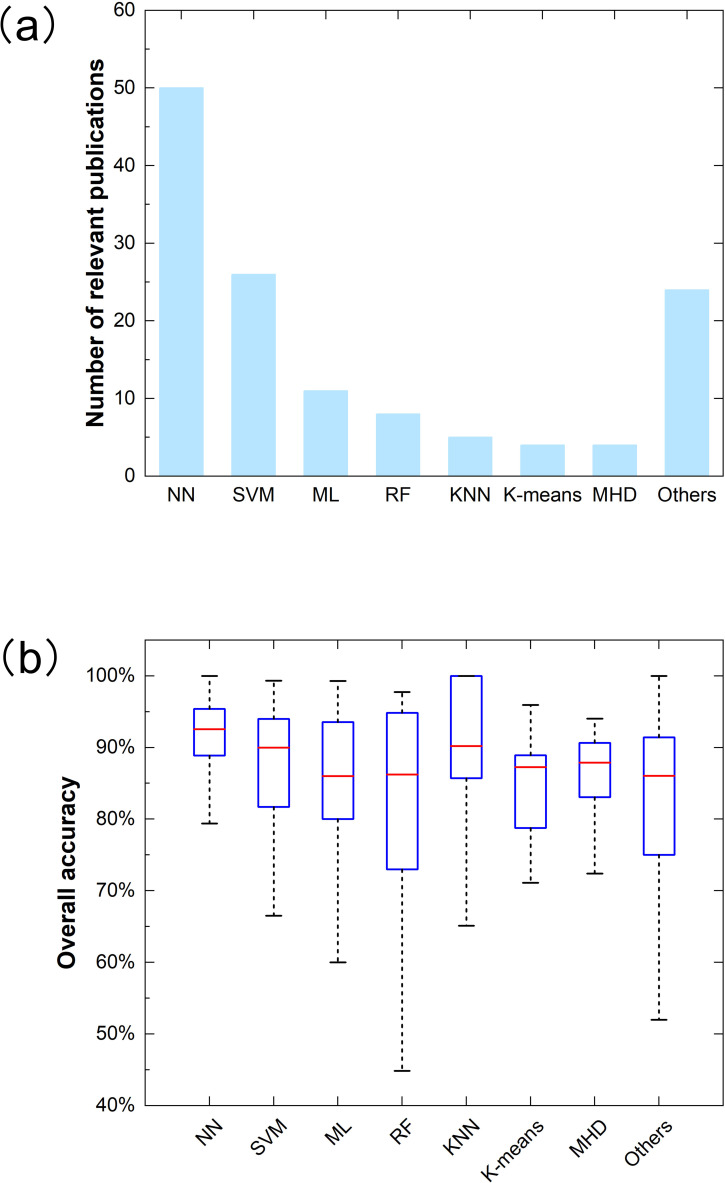
Distribution **(a)** and overall accuracy (OA) **(b)** for different classification methods in related publications.

NN, SVM, and ML are the most commonly used methods in related studies, with 50,26 and 11 studies, respectively (**Figure 12a**). And RF is next to the above, with eight studies. Consistent with the findings of Masoud Mahdianpari et al (Mahdianpari et al., 2020a), CNN models are generally more common in remote sensing applications than other deep learning methods (Kamilaris and Prenafeta-Boldú, 2018; Yao et al., 2017). The results show that the neural network method has succeeded in many applications compared to the conventional methods used in UAV remote sensing identification (Deng et al., 2020; Zhang et al., 2022). In addition, SVM, with its strong generalization ability and optimal solution and discrimination ability, also attracted the industry’s attention (Cervantes et al., 2020). There are only five studies on the K-nearest neighbor (KNN) algorithm.

The overall accuracy box plot of different methods is shown in **Figure 12b**). The median overall accuracy for all methods is higher than 85.00%. The median overall accuracy of NN is the highest, at 92.55%. Meanwhile, NN has lower variability than other methods. This shows that NN performs excellently in agricultural identification (Chew et al., 2020; Darwin et al., 2021). However, in most cases, artificial intelligence is still a “black box” with poor interpretability (Li et al., 2022b). Followed by KNN, its median accuracy was 90.19%. Maximum likelihood (ML) classification had the lowest median overall accuracy, about 86.00%. In addition, RF has the worst stability, probably because a small amount of training data will likely cause misclassification for the RF algorithm.

In addition, we analyzed the overall accuracy of various classification methods based on different sensors and study targets. It is worth noting that no further analysis was performed under the smaller sample size categories such as “invasive plant identification” and “cultivated land identification” in the study target. At the same time, the classification method with less than three samples and “Others” are ignored to ensure the relative reliability and reference of the analysis results.

**Figure 13:**
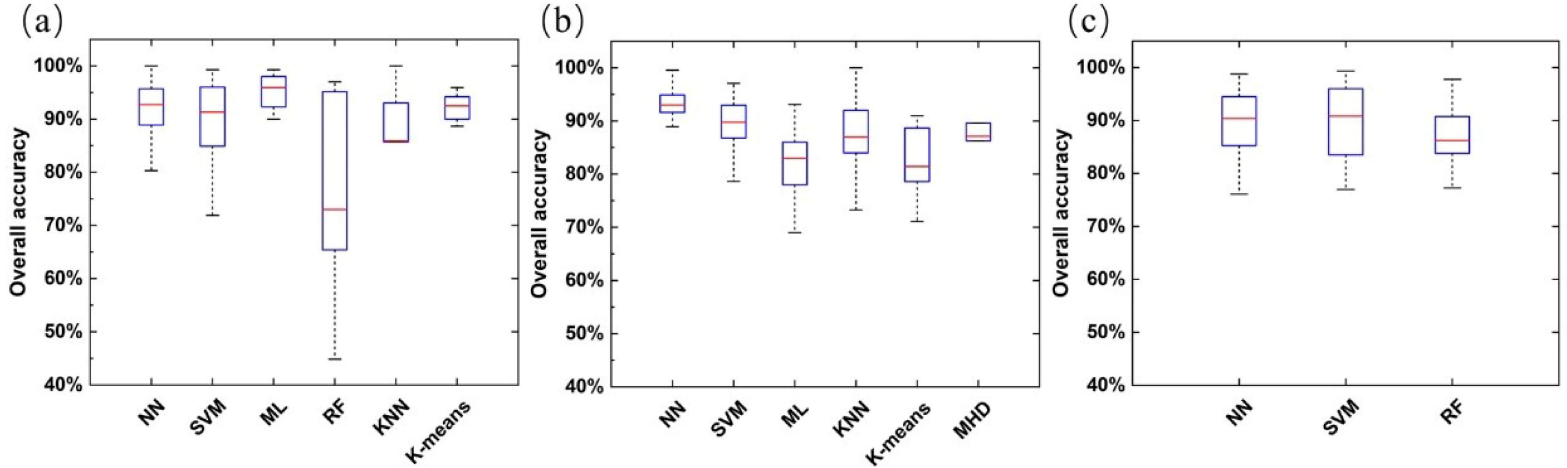
The overall accuracy (OA) distribution of classification methods under different sensor types [**(a)** RGB, **(b)** Multispectral, **(c)** hyperspectral].

**Figures 13a, b, c** show the overall accuracy distribution of the classification method under RGB, multispectral, and hyperspectral sensors, respectively. It can be seen that ML has the highest median overall accuracy of 95.93% in studies using RGB sensors, and the lower and upper whiskers extend from 90.00% to 99.30%. This may be because such a method is more suitable for multi-band data with fewer bands, which is one of the reasons for their wide application (Hogland et al., 2017; Sun et al., 2013). Therefore, the ML method, which is simple and easy to operate, may be more suitable for agricultural identification using RGB sensors. They were followed by NN ‘s median overall accuracy of 92.70%, which is unsurprising. Simultaneously, NN is also a method with the highest median overall accuracy in multi-spectral sensor applications, reaching 93.02%. The highest median overall accuracy of the remaining techniques is SVM, 89.75%. The K-means algorithm performs worst, with a median overall accuracy of only 81.44%. Therefore, in the research based on multi-spectral data, we can use more ISODATA algorithms (Li et al., 2020; Wang et al., 2011). The three main methods of research based on hyperspectral data are SVM, CNN, and RF. Among them, the median overall accuracy of SVM is the highest at 90.84%, while CNN’s is similar at 90.37%.

**Figure 14:**
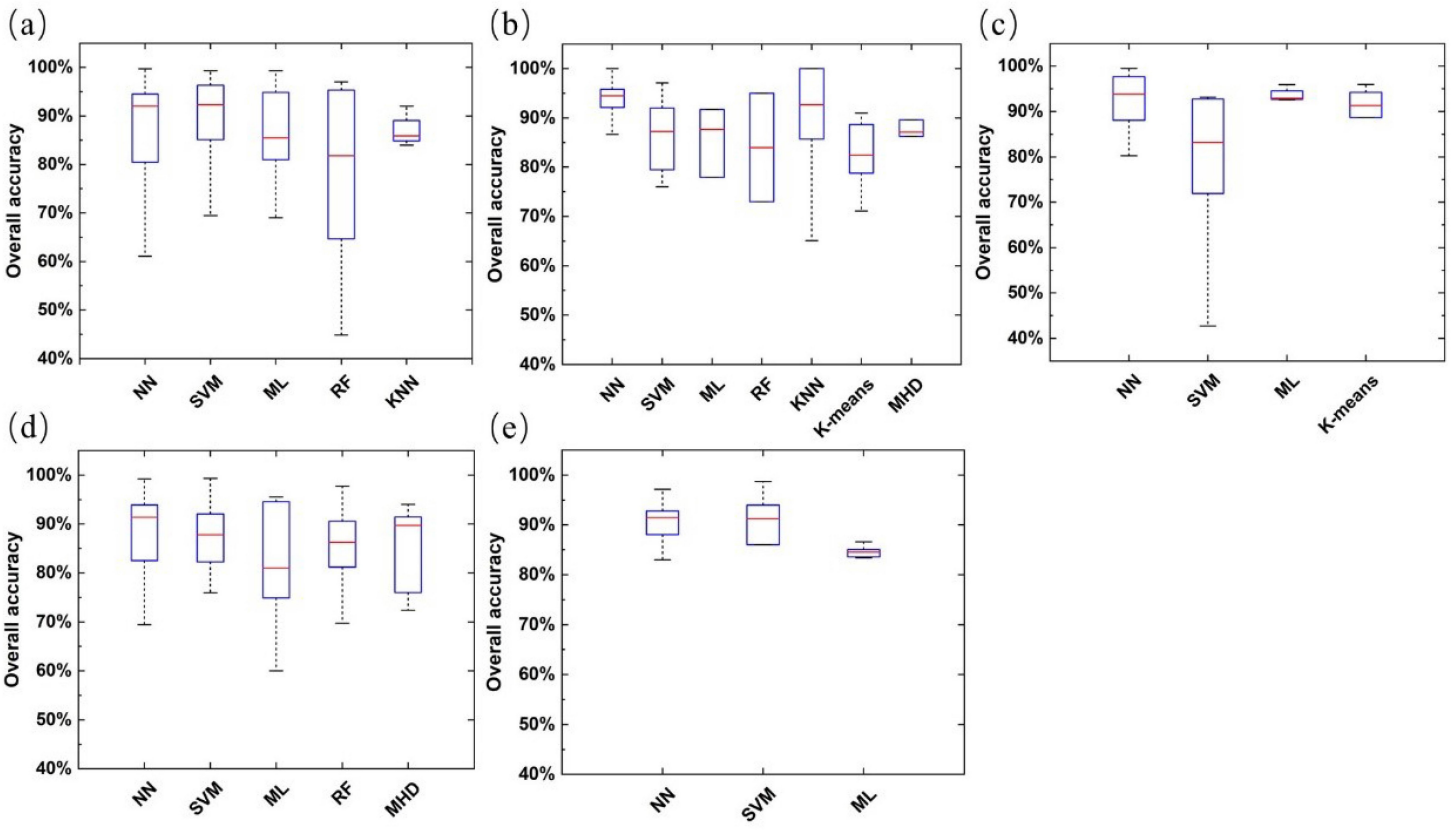
The overall accuracy (OA) distribution of classification methods under different study targets [**(a)** weed detection, **(b)** plant pests and diseases (vigor) recognition, **(c)** plant identification or counting, **(d)** crop identification or classification, **(e)** plant lodging identification].

**Figure 14** shows the overall accuracy of each method in research applications for different study targets. In addition to weed detection, NN still has the best identification performance among other study targets, with the highest median overall accuracy. This shows that neural networks, especially CNN, are increasingly favored by agricultural remote sensing practitioners and have achieved remarkable results with the popularity of deep learning algorithms. However, the best method for identification performance in related applications of weed detection is SVM. This may be because the study of weed identification is more of a two-category classification task (Mathur and Foody, 2008b); the specific reasons are also worth further exploration.

Our meta-analysis showed the advantages and disadvantages of numerous methods for vegetation classification, suggesting almost none of them is universally applicable (Du et al., 2012). Therefore, With the increasing demand for classification accuracy, multi-classification systems have gradually developed, which can integrate the advantages of multiple classification algorithms and overcome the limitations of a single algorithm (Ceamanos et al., 2010). Choosing the appropriate algorithm combination can improve the accuracy of classification results. Chen et al. (2017) introduced a multi-classifier system MCS_WV_AdaBoost, which integrates SVM, C4.5, and ANN with AdaBoost as the combining strategy. This system aimed to extract land use/cover information from a time series of remote sensing images from 1987 to 2015 at an average interval of 3 years, ultimately leading to enhanced accuracy.

## 5 Recommendations and prospects

Through the comprehensive analysis of agricultural identification based on UAV remote sensing in recent years, we found that the application of related fields has considerably developed. Still, the full potential in some areas has yet to be tapped. This section aims to point out some research challenges and prospects for future directions:

### 5.1 Feature selection and extraction

In the problem of vegetation classification based on UAV remote sensing, due to the similar spectral information of vegetation itself, it is easy to the phenomenon of “same object different spectrum” or “foreign object same spectrum”, which affects the classification accuracy. Almost all plant life is affected by seasonal changes, the seasonal phases or dynamics of this plant, called phenology (Kattenborn et al., 2021). This means that changes in plant characteristics with the phenological stages can be reflected in the spectral data (Zhou et al., 2023). Using phenological information to assist species classification has strong operability (Weisberg et al., 2021). Different species have different seasonal effects, and the specificity of their phenological characteristics can be used as an essential reference index for classification. The work of Torres-Sánchez et al. meticulously evaluated the impact of spatial and spectral resolution of UAV imagery in a multi-temporal study (Torres-Sánchez et al., 2014).

Subtle differences and complex relationships between many vegetation species require more precise correlation features (Shahi et al., 2022). With the improvement of spatial resolution of remote sensing images, many features are used for vegetation classification, such as spectral features (Torres-Sánchez et al., 2015), color features, and spatial features (Suarez et al., 2013). Stroppiana et al. (2018) used the multi-spectral spectral data of UAV to monitor weeds and added a variety of vegetation indices and texture features. The results showed that the classification based on the vegetation index had a good effect, and the overall accuracy was 96.50%. However, it should be noted that conventional vegetation indices like NDVI may reach saturation in dense vegetation canopies (LAI > 3), limiting their sensitivity to subtle variations in high-biomass conditions (Gitelson, 2004). To address this, recent studies have proposed: (1) enhanced vegetation indices (e.g., EVI2) that maintain linearity under high LAI conditions, and (2) the integration of texture metrics with narrow-band indices derived from hyperspectral sensors, particularly in the red-edge region (700-750 nm), which shows improved discrimination capability for dense vegetation (Appice and Malerba, 2019).

However, blindly adding more features to the classification process is likely to cause a large amount of data redundancy and even cause the problem of “dimension disaster” (Zhang et al., 2017). Especially for hyperspectral sensors, detecting of numerous spectral bands with strong correlations between adjacent bands has posed particular challenges for data processing and analysis (Jia et al., 2011). Hence, feature selection and feature extraction are vital parts of UAV vegetation classification, and the effective use of multiple features is the key to improving classification accuracy (Fu et al., 2017). This helps prevent any loss in accuracy within the vegetation classification model due to data redundancy. Feature selection involves directly choosing specific spectral feature variables from the original variables for modeling. In contrast, feature extraction entails extracting information from the original variables to obtain a new set of variables based on mathematical relationships among spectral variables (Xiao et al., 2022). Especially in the current research, the spectral feature extraction of grassland vegetation is still concentrated in the RGB band, and the spectral features of multispectral and hyperspectral are worthy of in-depth exploration.

### 5.2 Expansion of data sets and optimization of deep learning algorithms

Our research demonstrates that convolutional neural networks (CNNs), the most widely used method among deep learning algorithms, offer significant advantages in agricultural UAV remote sensing applications. However, training CNNs requires a substantial amount of labeled data, which poses a significant challenge in agricultural applications (Ghanbari et al., 2021). To address this challenge, future efforts should actively explore advanced data augmentation techniques, such as data synthesis methods based on Generative Adversarial Networks (GANs), to generate realistic virtual agricultural images and expand the training dataset (Hao et al., 2023). Additionally, the use of transfer learning strategies, where models pre-trained on large-scale general image datasets are adapted to specific agricultural tasks, can leverage the learned general features and reduce reliance on large-scale, agriculture-specific datasets, thereby improving the stability and accuracy of models in agricultural UAV remote sensing identification tasks.

### 5.3 The integration of edge computing and real-time vegetation identification decision-support systems

In UAV-based vegetation identification studies, how to rapidly transform the large amounts of remotely sensed data collected in real-time into effective vegetation information is crucial for enhancing accuracy and response speed. Currently, images and data generated by UAV remote sensing technology usually need to be processed through the cloud. However, this often leads to delays, affecting the real-time nature of vegetation identification and subsequent decisions. To address this issue, edge computing technology can be utilized to perform local data processing and vegetation identification directly on drones or ground stations (Li et al., 2024). Through an edge computing platform, efficient image processing and machine learning models can be used to identify vegetation types, health status, and growth stages in real-time on-site, without the need to transmit large amounts of data to the cloud. This approach not only significantly enhances processing speed but also enables rapid decision-making in urgent situations, such as precise livestock management, targeted irrigation, fertilization, or pest control.

### 5.4 Breakthrough in multi-source data fusion technology

At present, all kinds of remote sensing data products have advantages and disadvantages, so multi-source fusion has become one of the directions of exploration in the current and future fields. However, during the fusion process, different spatiotemporal scales of different datasets make effective information interaction challenging (Li et al., 2022a). Furthermore, the similarity of vegetation spectral characteristics is a pervasive problem. Classification methods that rely solely on remote sensing spectral data yield limited results in some instances. To break through these bottlenecks, innovative fusion methods should be developed in the future, such as developing end-to-end fusion models based on deep learning, mining fusion features from raw multi-source data, and combining high-resolution details of drone images with the macro vision of satellite data. At the same time, multi-modal data is incorporated, integrating not only remote sensing data but also geographical, meteorological, soil, and other types of information to provide a more comprehensive basis for identification (Hong et al., 2021).

### 5.5 Establishment of a standardized process for vegetation identification using UAV remote sensing

While UAV remote sensing technology holds immense potential for agricultural identification, there is still a notable absence of transparent standardized processes in specific research and application contexts. For example, determining the optimal flight altitude, spatial resolution, and sensor selection under different environmental and terrain conditions to balance flight efficiency and identification accuracy remains a technical challenge. The choice of sensors, such as multispectral, hyperspectral, or LiDAR, significantly impacts the quality and type of data captured, influencing the accuracy of vegetation classification and health assessment. Additionally, selecting an appropriate spatial resolution based on the specific requirements of vegetation identification—whether it is for large-scale crop monitoring or fine-scale health analysis—is critical for achieving optimal results. In the future, a standardized system should be established that encompasses various aspects such as drone selection, flight parameter settings, sensor specifications, data collection protocols, and data processing procedures (Wang et al., 2024). Through a large number of experiments and data analyses, the optimal parameter combinations for different agricultural scenarios, including sensor type and spatial resolution, will be determined (Lukas et al., 2022). An intelligent UAV flight and data acquisition system will be developed to achieve one-click operation, lower the operation threshold, and improve the efficiency and reliability of UAV remote sensing in agricultural applications.

### 5.6 Challenges and future development directions of identifying vegetation in grassland

Identifying the vegetation in grassland is an important direction in the application of UAV remote sensing in agriculture. However, the existing technologies are mainly focused on the identification of agricultural crops, and there are still big challenges for the identification of grassland species whose growth is not easy to control. In the future, further research is needed to develop classification algorithms tailored for grassland vegetation, taking into account factors such as growth characteristics, community structure, and others, in order to create a model better suited for herbaceous plants (Lyu et al., 2022). This not only promotes the coordinated development of agriculture and animal husbandry but also provides a scientific basis for the ecological protection and sustainable use of grasslands, thereby facilitating the rational development and management of grassland resources.

## 6 Conclusion

Agriculture identification in remote sensing has recently drawn widespread attention and obtained fine performances. This paper provides a comprehensive overview of vegetation identification using UAV remote sensing data. Based on information provided by 79 references, this study established a database of UAV-based agricultural identification processes and conducted meta-analyses to showcase the performance and trends of these studies.

Findings from our meta-analysis indicate that since 2016, the use of drones in agricultural identification applications has shown an increasing trend, and its growth rate is also rising. Further analysis indicates that identification based on UAV remote sensing is widely used in plant pest (vitality) identification, plant identification or counting, and weed identification. In addition, RGB is the sensor type with the highest overall accuracy and the most muscular stability. The medium spatial resolution remote sensing image is the most commonly used data source. With the improvement of spatial resolution, the potential to achieve higher overall accuracy is also increasing. Among the various methods, NN and SVM are the most commonly used. In particular, neural networks have shown the best performance in vegetation identification based on UAV and have garnered widespread attention in recent years. While this study has made significant contributions, it also acknowledges limitations such as regional data biases and the inherent constraints of meta-analysis, suggesting that future research should focus on mitigating these biases to enhance the accuracy and universality of the analysis.

In conclusion, this review analyzed the research directions and progress in vegetation identification using UAV remote sensing, offering suggestions and prospects for future development. Although existing technologies have greatly enhanced vegetation identification and monitoring, there is still further need for improvement in the technical support for grassland remote sensing identification and management. By overcoming the limitations of current research, we aim to enhance the accuracy and reliability of agricultural remote sensing, ultimately supporting better agricultural management and promoting sustainable development.
